# Glymphatic system impairment in primary angle-closure glaucoma: A cross-sectional study of disease severity

**DOI:** 10.1097/MD.0000000000048565

**Published:** 2026-05-01

**Authors:** Zhang Zhao, Dan Liu, Xianjun Zeng, Tao You, Fei Xiong

**Affiliations:** a Department of Otolaryngology, Hubei No. 3 People’s Hospital of Jianghan University, Wuhan, China; b Department of Radiology, Hubei Cancer Hospital, Tongji Medical College, Huazhong University of Science and Technology, Wuhan, China; c Department of Radiology, The First Affiliated Hospital of Nanchang University, Jiangxi Province Medical Imaging Research Institute, Nanchang, China; d Department of Radiology, General Hospital of Central Theater Command, Wuhan, China.

**Keywords:** diffusion tensor image, glaucoma, glymphatic system

## Abstract

The glymphatic system plays a critical role in brain waste clearance and has been implicated in neurodegenerative diseases. Primary angle-closure glaucoma (PACG) is increasingly recognized as a neurodegenerative disorder extending beyond the eye. This study aimed to investigate glymphatic system function in patients with PACG using diffusion tensor imaging analysis along the perivascular space (DTI-ALPS). This cross-sectional study included patients with PACG and age- and sex-matched healthy controls. All participants underwent magnetic resonance imaging, including diffusion tensor imaging. The DTI-ALPS index was calculated to assess glymphatic function. Group differences and correlations with disease severity were analyzed. Patients with PACG showed a significantly lower whole-brain DTI-ALPS index compared with healthy controls (1.50 ± 0.14 vs 1.60 ± 0.15, *P* = .030; Cohen *d* = 0.63). Lower DTI-ALPS values were significantly correlated with worse visual field mean deviation and thinner retinal nerve fiber layer thickness (*P* < .05). Glymphatic system function appears to be impaired in PACG and is associated with disease severity. These findings provide further evidence supporting the concept of glaucoma as a neurodegenerative disease involving the central nervous system.

## 1. Introduction

Glaucoma is a predominant cause of irreversible blindness worldwide and represents a major public health burden.^[1]^ Primary angle-closure glaucoma (PACG) is associated with rapid disease progression and severe visual impairment.^[1]^ Although glaucoma has traditionally been considered an ocular disease characterized by elevated intraocular pressure (IOP) and optic nerve damage, increasing evidence suggests that it shares pathological features with neurodegenerative disorders.^[2–8]^

Neuroimaging studies have demonstrated structural and functional alterations in multiple brain regions in patients with glaucoma, extending beyond the visual pathway.^[7]^ These findings support the concept that glaucoma involves widespread neurodegeneration rather than isolated optic nerve injury.

The glymphatic system is a recently described brain-wide clearance pathway that facilitates the removal of metabolic waste products through perivascular spaces.^[9–11]^ Glymphatic dysfunction has been implicated in the pathogenesis of several neurodegenerative diseases, including Alzheimer disease and Parkinson disease.^[12–15]^ Given the shared pathological features between glaucoma and neurodegenerative disorders, impaired glymphatic clearance may also play a role in glaucoma-related neurodegeneration.

Diffusion tensor imaging analysis along the perivascular space (DTI-ALPS) has emerged as a noninvasive imaging method for assessing glymphatic system activity in humans.^[16–18]^ Altered DTI-ALPS indices have been reported in various neurological conditions^[19–22]^; however, glymphatic function in PACG remains largely unexplored.

We hypothesized that brain glymphatic function is impaired in PACG and that reduced glymphatic activity is associated with disease severity.

## 2. Materials and methods

### 2.1. Subjects

The present study was approved by the Institutional Review Board of First Affiliated Hospital, Nanchang University (approval number: No. IIT2024-283), and complied with the principles of the Declaration of Helsinki. All participants provided informed consent before taking part in the study. Thirty-one PACG patients and 25 age- and sex-matched healthy controls (HCs) were enrolled in the study. The PACG patients were recruited from both the inpatient and outpatient clinics of The First Affiliated Hospital, Nanchang University. Concurrently, age- and sex-matched HCs were recruited from the local community through advertisements. The diagnosis of glaucoma was established in accordance with the diagnostic criteria of the Primary Angle Closure Preferred Practice Pattern Guidelines of the American Academy of Ophthalmology.^[23]^

The inclusion criteria of PACG patients are listed as follows: right-handed, age 20 to 70 years old, IOP > 21 mm Hg, narrow anterior chamber angle, and glaucomatous visual field defects. Exclusion criteria were the following: secondary glaucoma, other ocular diseases (e.g., high myopia, strabismus, iridocyclitis, macular degeneration), history of neuropsychiatric disorders, and magnetic resonance imaging (MRI) contraindications. Systemic comorbidities and medication use were not explicitly controlled in this study.

### 2.2. Ophthalmic examination

An ophthalmologist with 15 years of experience in glaucoma examined all the patients. The ophthalmological examination included IOP measurement, visual fields assessment utilizing the Humphrey Field Analyzer II, and retinal nerve fiber layer (RNFL) thickness, vertical cup-to-disc ratio (VCDR), and horizontal cup-to-disc ratio (HCDR) measurement utilizing high-definition optical coherence tomography (OCT).

### 2.3. MRI data acquisition

All MRI scans were conducted on a 3.0-Tesla Siemens Trio MRI scanner using an 8-channel phased-array head coil. T1-weighted images were obtained through 3D MPRAGE sequence with the consequent scan parameters: repetition time/echo time = 1900/2.26 ms, flip angle = 9°, field of view = 240 mm × 240 mm, slice thickness/gap = 1.0/0 mm, and 176 sagittal slices. The diffusion-weighted images were acquired through a single-shot spin-echo echo planar imaging sequence with the consequent scan parameters: repetition time/echo time = 8000/89 ms, field of view = 250 mm × 250 mm, 65 axial slices, slice thickness = 2.0 mm, 64 gradient directions using with b = 1000 s/mm^2^, and a b value = 0 (b0) image.

### 2.4. DTI-ALPS processing

The preprocessing of DTI images involved several steps: denoising, Gibbs ringing removal, distortion and motion artifacts correction, and bias field correction. We generated diffusion maps of directions in the x-axis, y-axis, z-axis, and fractional anisotropy (FA) maps using MRtrix3.^[24]^

The FA and diffusion maps along the x-axes, y-axes, and z-axes were generated using the FSL command line “dtifit.” Each subject’s FA map was coregistered to the JHU-ICBM-FA template. The transformation matrices from this coregistration were then applied to all diffusivity maps with FSL command line “flirt.” The association and projection fibers at the lateral ventricle body level based on the ICBM-DTI-81 atlas were recognized as the superior longitudinal fasciculus (SLF) and the superior corona radiata (SCR), respectively. The regions of interests with 5 mm diameter spheres were created automatically within the bilateral SLF and SCR areas, and the center coordinates were designated as below: left SLF (128, 110, 99), left SCR (116, 110, 99), right SLF (51, 110, 99), and right SCR (64, 110, 99) on the JHU-ICBM-FA template.

We record diffusion values in directions of the x-axes, y-axes, and z-axes of both SLF and SCR, denoted as Dxx-association, Dyy-association, Dzz-association, Dxx-projection, Dyy-projection, and Dzz-projection. The DTI-ALPS measurement was then estimated as (mean [Dxx-projection, Dxx-association]/mean [Dyy-projection, Dzz-association]). Three DTI-ALPS measurements were calculated: left hemisphere, right hemisphere, and whole-brain DTI-ALPS, respectively.

### 2.5. Statistical analysis

The participants’ demographics, clinical, and diffusion characteristics were presented utilizing means, medians, or proportions, and standard deviation or interquartile range as suitable. All the analyses were performed utilizing R software (version 4.2.3). The normal distribution was assessed utilizing the Shapiro–Wilk test. Group comparisons of demographics, clinical, and diffusion characteristics were conducted utilizing *t* tests, Mann–Whitney *U* tests, or χ^2^ tests as suitable.

We also assessed the relationships between DTI-ALPS measurements and several clinical indicators using Pearson correlation and false discovery rate correction in the PACG group. These indicators include disease duration, IOP, RNFL, HCDR, and VCDR.

Given the exploratory nature of this neuroimaging study and the lack of established effect size estimates for glymphatic function in PACG, a formal a priori sample size calculation was not performed. Statistical significance was set at *P* < .05.

## 3. Results

### 3.1. Demographics and clinical information

Thirty-one PACG patients and 25 HCs were recruited in the present study. After ruling out 3 PACG patients and 2 HCs, due to incomplete clinical data and low image quality, the final analyses included 28 PACG patients (11 males/17 females; age, 53.0 ± 10.4 years) and 23 HCs (8 males/15 females; age, 51.0 ± 10.7 years). The 2 groups showed no difference in terms of age and gender. The IOP, RNFL, HCDR, and VCDR were available to 28 patients with PACG. The above clinical metrics were not collected in HCs. Participant demographics and clinical information are described in Table 1.

**Table 1:** Demographic and clinical characteristics of PACG patients and HC.

Characteristics	PACG (n = 28)	HC (n = 23)	*t*/χ^2^	*P*
Age (yr)	52.96 ± 10.44	51 ± 10.65	0.662	.511
Gender (male)	11 (39.2%)	8 (36.3%)	0.045	.833
IOP_L (mm Hg)	25.07 ± 12.11	NA		
IOP_R (mm Hg)	27.96 ± 13.48	NA		
Mean IOP (mm Hg)	26.51 ± 8.02	NA		
RNFL_L (μm)	82.57 ± 22.43	NA		
RNFL_R (μm)	75.14 ± 19.73	NA		
HCDR_L	0.70 ± 0.16	NA		
HCDR_R	0.71 ± 0.15	NA		
VCDR_L	0.67 ± 0.20	NA		
VCDR_R	0.70 ± 0.17	NA		
Duration of illness (mo)	14.93 ± 23.41	NA		

HC = healthy controls, HCDR = horizontal cup-to-disc ratio, HCDR_L = horizontal cup-to-disc ratio, left eye, HCDR_R = horizontal cup-to-disc ratio, right eye, IOP = intraocular pressure, IOP_L = intraocular pressure, left eye, IOP_R = intraocular pressure, right eye, PACG = primary angle-closure glaucoma, RNFL = retinal nerve fiber layer, RNFL_L = retinal nerve fiber layer, left eye, RNFL_R = retinal nerve fiber layer, right eye, VCDR = vertical cup-to-disc ratio, VCDR_L = vertical cup-to-disc ratio, left eye, VCDR_R = vertical cup-to-disc ratio, right eye.

### 3.2. DTI-ALPS index

**Figure 1.**
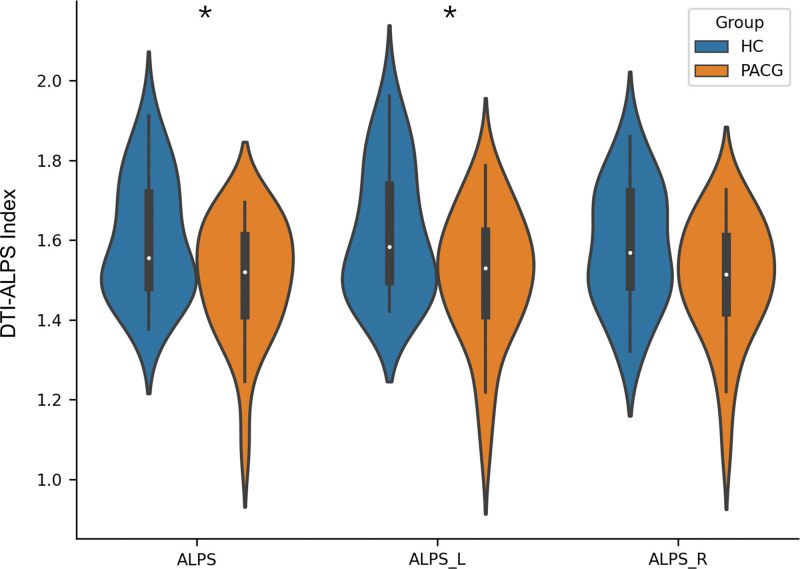
Comparisons of the DTI-ALPS index. Comparisons of the DTI-ALPS index in the left, right hemisphere and whole-brain between the PACG and HCs groups. *Indicates *P* value < .05. ALPS = along the perivascular space, DTI-ALPS = diffusion tensor imaging analysis along the perivascular space, HC = healthy controls, PACG = primary angle-closure glaucoma.

Compared with HCs, PACG patients exhibited a higher diffusion value in the z-axis of the left SLF (0.41 ± 0.06 vs 0.37 ± 0.04; *P* = .028; Cohen *d* = 0.77). PACG patients exhibited a lower whole-brain DTI-ALPS measurement compared with HCs (1.50 ± 0.14 vs 1.60 ± 0.15; *P* = .030; Cohen *d* = 0.63). In addition, the left-hemisphere DTI-ALPS measurement was also significantly lower in PACG patients (1.51 ± 0.16 vs 1.61 ± 0.16; *P* = .030; Cohen *d* = 0.63). Although the right-hemisphere DTI-ALPS measurement tended to decrease in PACG patients compared with HCs, the difference did not reach statistical significance (1.50 ± 0.15 vs 1.58 ± 0.15; *P* = .061). These findings are summarized in Table 2 and illustrated in Figure 1.

**Table 2:** Group comparisons of ALPS.

Features	PACG (n = 28)	HC (n = 23)	*F*	*P*	Cohen *d*
ALPS	1.50 ± 0.14	1.60 ± 0.15	5.003	.03	0.63
ALPS_L	1.51 ± 0.16	1.61 ± 0.16	5.004	.03	0.63
ALPS_R	1.50 ± 0.15	1.58 ± 0.15	3.681	.06	0.53
x_projection_L (×10^−3^ mm^2^/s)	0.62 ± 0.06	0.62 ± 0.07	0.282	.59	0.01
x_association_L (×10^−3^ mm^2^/s)	0.73 ± 0.06	0.74 ± 0.05	0.278	.6	0.18
y_projection_L (×10^−3^ mm^2^/s)	0.48 ± 0.07	0.47 ± 0.05	0.303	.59	0.16
z_association_L (×10^−3^ mm^2^/s)	0.41 ± 0.06	0.37 ± 0.04	5.139	.03	0.77
x_projection_R (×10^−3^ mm^2^/s)	0.58 ± 0.06	0.60 ± 0.07	0.76	.39	0.31
x_association_R (×10^−3^ mm^2^/s)	0.68 ± 0.06	0.68 ± 0.05	0.091	.77	<0.001
y_projection_R (×10^−3^ mm^2^/s)	0.47 ± 0.08	0.45 ± 0.05	0.762	.39	0.29
z_association_R (×10^−3^ mm^2^/s)	0.38 ± 0.06	0.36 ± 0.05	1.535	.22	0.36

ALPS = along the perivascular space, HC = healthy control, L = left, PACG = primary angle-closure glaucoma, R = right.

### 3.3. Relationships of DTI-ALPS and clinical characteristics in PACG

**Figure 2.**
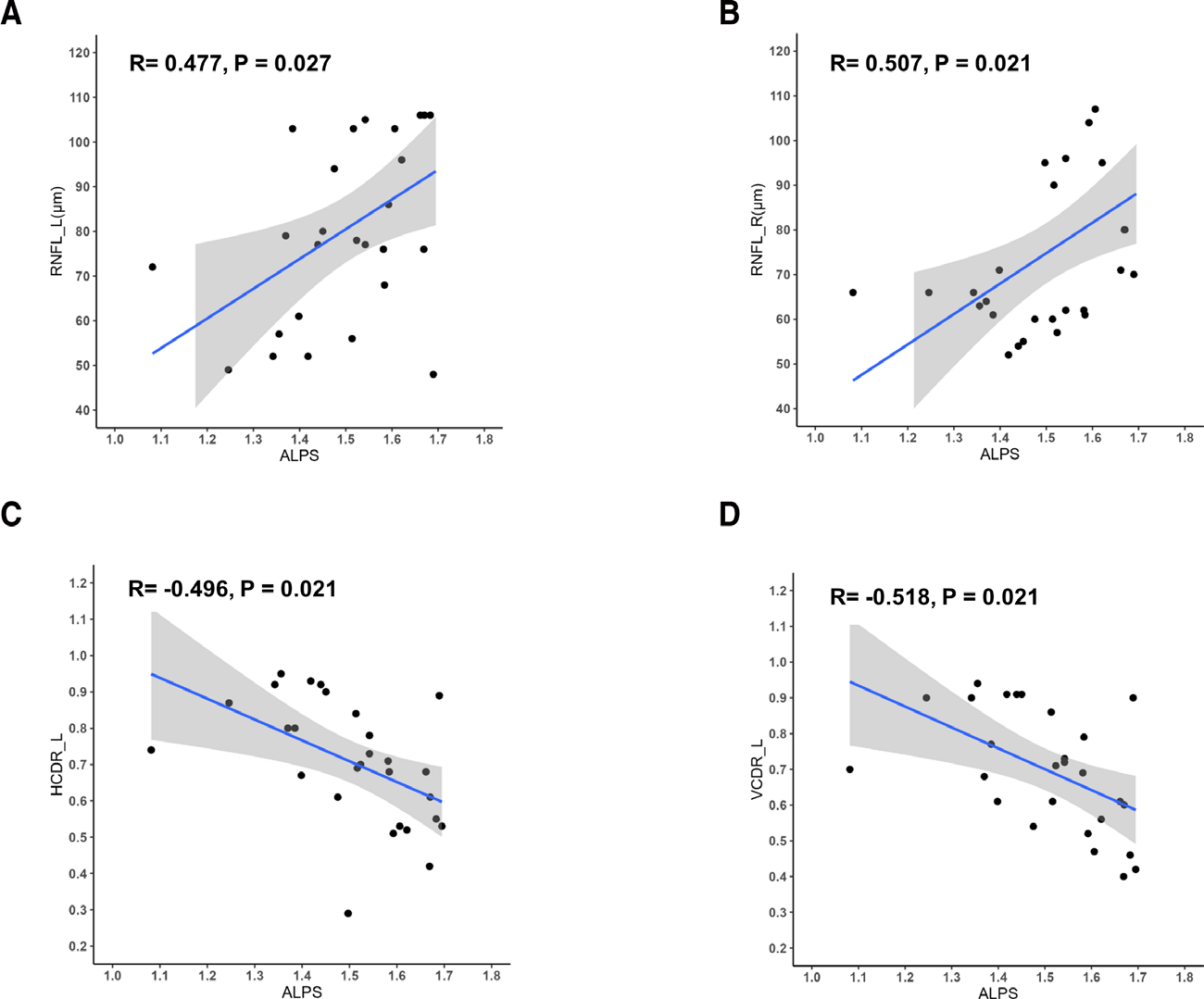
Correlations among the whole-brain DTI-ALPS index and clinical characteristics in PACG group. (A) Correlation of the whole-brain DTI-ALPS index with RNFL in the left eye. (B) Correlation of the whole-brain DTI-ALPS index with RNFL in the right eye. (C) Correlation of whole-brain DTI-ALPS with HCDR in the left eye. (D) Correlation of the whole-brain DTI-ALPS index with VCDR in the left eye. Pearson correlation analyses were used all the above. ALPS = along the perivascular space, DTI-ALPS = diffusion tensor imaging analysis along the perivascular space, HCDR_L = horizontal cup-to-disc ratio, left eye, PACG = primary angle-closure glaucoma, RNFL_L = retinal nerve fiber layer, left eye, RNFL_R = retinal nerve fiber layer, right eye, VCDR_L = vertical cup-to-disc ratio, left eye.

In individuals diagnosed with PACG, a lower whole-brain DTI-ALPS measurement was significantly associated with a higher HCDR in bilateral eyes (left eye: *r* = −0.496, *P* = .007; right eye: *r* = −0.402, *P* = .034), an increased VCDR in the left eye (*r* = −0.518, *P* = .006), as well as a reduced RNFL thickness in both eyes (left eye: *r* = 0.477, *P* = .012; right eye: *r* = 0.507, *P* = .006). Contrary to expectations, no significant relationship existed between the DTI-ALPS measurements and IOP (left eye: *P* = .884; right eye: *P* = .552). However, following multiple testing correction (false discovery rate), robust correlations persisted between the global DTI-ALPS measurement and RNFL thickness in bilateral eyes (left eye: *r* = 0.507, *P* = .032; right eye: *r* = 0.477, *P* = .027), as well as with VCDR and HCDR in left eye (*r* = −0.496, *P* = .021; *r* = −0.518, *P* = .021) as depicted in Table 3 and illustrated in Figure 2.

**Table 3:** Relationships between ALPS and clinical variables in patients with primary angle-closure glaucoma.

Feature	Clinical variables	*R*	*P*	*P* (FDR corrected)
ALPS	Duration of illness (mo)	−0.109	.578	.65
ALPS	IOP_L (mm Hg)	0.029	.884	.884
ALPS	IOP_R (mm Hg)	−0.117	.552	.65
ALPS	RNFL_L (μm)	0.477	.012	.027
ALPS	RNFL_R (μm)	0.507	.006	.021
ALPS	HCDR_L	−0.496	.007	.021
ALPS	HCDR_R	−0.402	.034	.061
ALPS	VCDR_L	−0.518	.006	.021
ALPS	VCDR_R	−0.322	.094	.141

ALPS = along the perivascular space, FDR = false discovery rate, HCDR = horizontal cup-to-disc ratio, HCDR_L = horizontal cup-to-disc ratio, left eye, HCDR_R = horizontal cup-to-disc ratio, right eye, IOP = intraocular pressure, IOP_L = intraocular pressure, left eye, IOP_R = intraocular pressure, right eye, RNFL = retinal nerve fiber layer, RNFL_L = retinal nerve fiber layer, left eye, RNFL_R = retinal nerve fiber layer, right eye, VCDR = vertical cup-to-disc ratio, VCDR_L = vertical cup-to-disc ratio, left eye, VCDR_R = vertical cup-to-disc ratio, right eye.

## 4. Discussion

In the present investigation, we explored the functionality of the glymphatic system within a cohort of PACG subjects by employing a noninvasive method termed DTI-ALPS. Our findings revealed a pronounced disruption in glymphatic activity among PACG patients compared to HCs. In addition, our analysis uncovered a significant correlation between the deterioration of glymphatic function and the exacerbation of clinical indicators, including RNFL thickness, HCDR, and VCDR within the PACG group.

Glaucoma, a predominant cause of nonreversible blindness worldwide, compels an in-depth inquiry into its complex pathophysiological underpinnings – an endeavor both critical and formidable.^[1,25]^ Emerging studies have posited the presence of an ocular glymphatic system, akin to that in the brain, within the eye and optic nerve.^[7,14,26]^ The dysfunction of the ocular glymphatic clearance mechanism has been documented in murine glaucoma models.^[7]^ Considering the optic nerve as a white matter tract extending from the brain, and noting the similarities between these 2 components of the central nervous system, it is reasonable to hypothesize that glymphatic pathways facilitating interstitial fluid and cerebrospinal fluid exchange may exist across both regions.^[27]^ A potential interconnection between the brain glymphatic pathway and the optic nerve subarachnoid space could support cerebrospinal fluid–interstitial fluid exchange and waste clearance processes.

Our results indicate altered glymphatic-related diffusion characteristics in the brains of individuals with PACG. When considered together with previously reported abnormalities in the ocular glymphatic system, these findings suggest that impaired glymphatic transport may be associated with glaucomatous neurodegeneration. Rather than establishing a causal relationship, our results highlight a potential link between glymphatic dysfunction and neurodegenerative processes observed in PACG. In this context, altered glymphatic function may represent a shared feature underlying the reported association between glaucoma and other neurodegenerative conditions, such as Alzheimer disease, although this hypothesis requires further investigation.

The attenuation of RNFL thickness observed on OCT serves as direct clinical evidence of retrograde degeneration of retinal ganglion cells in PACG.^[28]^ OCT remains the predominant diagnostic modality for assessing structural damage in glaucoma; however, it has notable limitations. RNFL measurements are subject to a “floor effect,” whereby visual field deterioration may continue despite minimal detectable RNFL thinning, limiting its utility in advanced disease stages.^[29]^ In addition, ocular media opacities, such as dense cataracts or corneal scarring, may compromise OCT accuracy.^[30]^ Given the irreversible nature of glaucomatous damage, complementary biomarkers capable of reflecting central nervous system involvement may enhance disease evaluation. In this regard, the DTI-ALPS index may serve as a potential adjunctive imaging marker reflecting brain glymphatic-related changes in PACG. Furthermore, this metric could be valuable in future in vivo studies assessing emerging therapeutic strategies, including neuroprotective interventions.^[31,32]^

The efficiency of the glymphatic system is influenced by multiple factors, including aquaporin-4 channel distribution, arterial pulsatility, respiration, body posture, and arousal state.^[33]^ Given the limited evidence regarding aquaporin-4 alterations in PACG, and the relative uniformity of arterial pulsation, respiratory patterns, and scanning posture across participants, our interpretation focuses on arousal-related factors. Sleep disturbances are reportedly more prevalent in PACG patients than in the general population and include irregular sleep patterns, delayed sleep onset, circadian rhythm disruption, and daytime dysfunction.^[34,35]^ As glymphatic activity is known to be enhanced during sleep,^[11]^ sleep disturbances may negatively influence glymphatic clearance efficiency.^[36,37]^ While speculative, our findings support further investigation into sleep quality as a modifiable factor potentially associated with PACG progression.

This study represents an initial exploration of glymphatic system function in PACG patients; however, several limitations should be acknowledged. First, the cross-sectional design and relatively small sample size limit causal inference and generalizability, necessitating confirmation in larger longitudinal cohorts. Second, the DTI-ALPS index reflects diffusion characteristics primarily along the perivenous space at the lateral ventricle body level and therefore provides only an indirect and partial assessment of glymphatic activity. Consequently, interpretations of glymphatic dysfunction based on this metric should be made cautiously. Third, although HCs were confirmed to have no ophthalmologic disease history, clinical ophthalmic parameters such as IOP, RNFL thickness, HCDR, and VCDR were not collected in this group, which may limit comparative clinical interpretation.

In conclusion, our investigation demonstrates altered glymphatic-related diffusion characteristics in PACG patients using a noninvasive MRI-based approach. Moreover, these alterations were associated with established clinical indicators of disease severity. Collectively, these findings support the hypothesis that glymphatic dysfunction may be involved in PACG pathophysiology and underscore the need for further studies to elucidate its clinical and mechanistic significance.

## Acknowledgments

We thank all the participants in this study.

## Author contributions

**Methodology:** Zhang Zhao, Fei Xiong.

**Data curation:** Dan Liu, Xianjun Zeng, Tao You.

**Validation:** Dan Liu, Tao You, Fei Xiong.

**Conceptualization:** Xianjun Zeng, Fei Xiong.

**Software:** Fei Xiong.

**Supervision:** Fei Xiong.

**Writing – review & editing:** Dan Liu, Tao You, Fei Xiong.

**Writing – original draft:** Zhang Zhao.
